# Chemical Interference with Iron Transport Systems to Suppress Bacterial Growth of *Streptococcus pneumoniae*

**DOI:** 10.1371/journal.pone.0105953

**Published:** 2014-08-29

**Authors:** Xiao-Yan Yang, Bin Sun, Liang Zhang, Nan Li, Junlong Han, Jing Zhang, Xuesong Sun, Qing-Yu He

**Affiliations:** 1 Key Laboratory of Functional Protein Research of Guangdong Higher Education Institutes, Institute of Life and Health Engineering, College of Life Science and Technology, Jinan University, Guangzhou, China; 2 School of Pharmaceutical Sciences, Southern Medical University, Guangzhou, China; East Carolina University School of Medicine, United States of America

## Abstract

Iron is an essential nutrient for the growth of most bacteria. To obtain iron, bacteria have developed specific iron-transport systems located on the membrane surface to uptake iron and iron complexes such as ferrichrome. Interference with the iron-acquisition systems should be therefore an efficient strategy to suppress bacterial growth and infection. Based on the chemical similarity of iron and ruthenium, we used a Ru(II) complex R-825 to compete with ferrichrome for the ferrichrome-transport pathway in *Streptococcus pneumoniae*. R-825 inhibited the bacterial growth of *S. pneumoniae* and stimulated the expression of PiuA, the iron-binding protein in the ferrichrome-uptake system on the cell surface. R-825 treatment decreased the cellular content of iron, accompanying with the increase of Ru(II) level in the bacterium. When the *piuA* gene (SPD_0915) was deleted in the bacterium, the mutant strain became resistant to R-825 treatment, with decreased content of Ru(II). Addition of ferrichrome can rescue the bacterial growth that was suppressed by R-825. Fluorescence spectral quenching showed that R-825 can bind with PiuA in a similar pattern to the ferrichrome-PiuA interaction *in vitro*. These observations demonstrated that Ru(II) complex R-825 can compete with ferrichrome for the ferrichrome-transport system to enter *S. pneumoniae*, reduce the cellular iron supply, and thus suppress the bacterial growth. This finding suggests a novel antimicrobial approach by interfering with iron-uptake pathways, which is different from the mechanisms used by current antibiotics.

## Introduction

Iron is a critical nutrient for bacterial growth and survival, as a major determinant in the development of infection in host. However, the concentration of free iron in host is extremely low (<10^−18^ M). In order to acquire enough iron from their host environments, bacteria have developed highly specific and effective iron-acquisition systems located on the membrane surface [1]–[3]. Blocking or interfering with the iron-acquisition systems could disrupt bacterial iron homeostasis and thus suppress bacterial growth.

*Streptococcus pneumoniae* is a dangerous bacterium responsible for various life-threatening diseases including otitis media, septicemia, pneumonia and meningitis in immuno-compromised individuals [4], [5]. In *S. pneumoniae*, there are three ABC transporters as known iron-transport systems including PiaABC, PiuABC and PitABC, respectively responsible for the acquisition of heme, ferrichrome and ferric irons [6]–[9]. Heme, ferrichrome and ferric irons are firstly bound by lipoproteins PiaA, PiuA and PitA and transferred to the permeases PiaB, PiuB and PitB to go across the cell membrane using energy provided by the ATP hydrolysis through PiaC, PiuC and PitC, respectively. Since the lipoproteins PiaA, PiuA and PitA work as iron-receptors located on the cellular surface, they are the valuable targets for the design of novel antibacterial agents. It must be pointed out that Cheng *et al* have characterized a ferrichorme-binding PiaA protein encoded by gene SP_1032 in *S. pneumnoniae* TIGR4 (corresponding to gene SPD_0915 in *S. pneumoniae* D39) [9], which is actually the protein PiuA through sequence alignments.

Earlier investigations have suggested that bacteria obtain iron mainly in the forms of complexes, heme-iron and ferrichrome-iron compounds as for *S. pneumoniae*. In this study, we selected our previously synthesized Ru(II) complex R-825 to chemically resemble the heme/ferrichrome compounds. Based on the chemical similarity between Fe(III) and Ru(II), we expected that R-825 could compete with heme/ferrichrome for binding to PiaA/PiuA in *S. pneumoniae*. Our experiments showed that R-825 indeed inhibited the bacterial growth by competing with ferrichrome for PiuA binding. We verified that complex R-825 can get access into the bacterium through ferrichrome-transport systems, reducing the iron supply and thus suppressing the bacterial growth.

## Materials and Methods

### Materials

**Figure 1:**
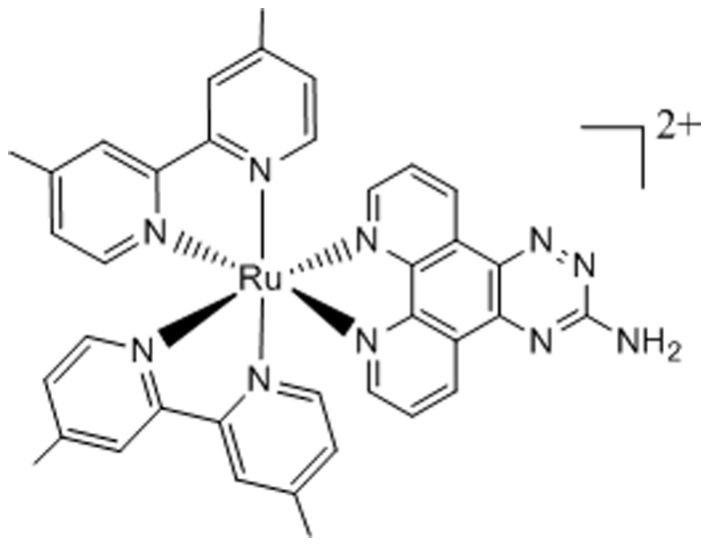
Structure of ruthenium(II) complex R-825 [10].

Ru(II) complex R-825 was synthesized according to the procedure with minor modifications as described in our previous publication, where the complex was defined as 1a [10]. The structure of R-825 is shown in Figure 1.

### Bacterial strains and growth conditions

Single gene deleted *piaA*- (SPD_1652), *piuA*- (SPD_0915) and *pitA*- (SPD_0226) mutant strains of *S. pneumoniae* D39 were constructed by long flanking homology-polymerase chain reaction (LFH-PCR) [11], [12], mutants were made by replacing *piaA*, *piuA* and *pitA* genes of D39 with gene encoding resistance to erythromycin (erm)[13]. Briefly, the 500 bp region upstream of *piaA* was amplified using primers piaA–P1 and piaA–P3, while the 500 bp region downstream of *piaA* was amplified using primers piaA–P2 and piaA–P4. The erythromycin gene was amplified using primers erm-F and erm-R. The three PCR fragments generated were joined together by overlap extension PCR using primers piaA–P1 and piaA–P2 to form approximately 2-kb final linear DNA construct the deletion fragment. Then, the linear DNA construct was used for homologous recombination and transformed into *S. pneumoniae* D39. Transformants were selected with 0.25 µg/mL erythromycin selection agar plates, mutants were confirmed by PCR and Western blotting. The *piuA*- and *pitA*- mutant strains were constructed using a similar method. All mutations were stable after six sequential passages in Todd-Hewitt broth supplemented with 0.5% yeast extract (0.5% THY) without antibiotic selection.

These mutant strains together with wild-type *S. pneumoniae* D39 were cultured at 37°C and 5% CO_2_ in 0.5% THY or on Columbia agar added with 5% sterile defibrinated sheep blood purchased from Ruite company (Guangzhou, China) in which all animal experiment procedures were conducted in strict accordance with the recommendations in the Guide for the Care and Use of Laboratory Animals of the National Institutes of Health. Disc susceptibility assays were performed as described by CLSI guidelines [14]. In short, filter paper disks (5 mm in diameter) containing 50 µg of R-825 were placed on the plates (10^7^ bacteria/plate), and inhibition zones were examined after 24 h of incubation at 37°C. Tests using gentamicin as the control were carried out in parallel.

### Minimal inhibitory concentration (MIC) assay

R-825 was dissolved in water to a concentration of 10 mg/mL (12 mM). The MIC assays were carried out in triplicate by using the standard micro-dilution method [14]. Different concentrations of R-825 with 7.5, 15, 30, and 60 µM were added to diluted media containing 10^6^ bacteria/mL, followed by incubation in 24-well plates at 37°C for 24 h. The concentration of R-825 corresponding to the well with no visible bacteria growth (OD_600_ < 0.1) was taken as the MIC.

### Cytotoxicity assay

The human alveolar epithelial cell line A549 was cultivated in DMEM media with 10% fetal bovine serum (FBS) at 37°C in a humidified atmosphere of 5% CO_2_. The cytotoxicity of R-825 in A549 cells was assessed using LDH cytotoxicity assay kit by the following procedure as described in the manufacture's manual. Briefly, 5×10^3^ A549 cells were seeded into a sterile 96-well plate in triplicate, incubated for 24 h without R-825, followed by a 48 h incubation in the dark with different amounts of R-825 (30, 60, 120, 240 and 480 µM). At the end of the incubation, the cell plate was centrifuged at 400 *g* for 5 min, then 120 µL of the media from each well was transferred to a new plate, and 60 µL of LDH mixture was added to the supernatant and incubated for 30 min in dark at room temperature. Cytotoxicity was assessed by monitoring the absorbance at 490 nm in a microplate reader. Whole cell lysate was used as a positive control.

### ICP-MS analysis

Sample preparation for ICP-MS was conducted as a previously described method [15]. Briefly, bacteria were grown in 0.5% THY in the presence or absence of R-825 with sub-MIC concentrations at 37°C and then harvested when the optical density at 600 nm (OD_600_) reached 0.5–0.6. Cells were pelleted by centrifugation at 8,000 *g* at 4°C for 10 min, and then washed three times with 1×PBS that had been treated with chelex-100 resin. Subsequently, the wet cell pellets were dried using a Scanvac Freeze Dryer (Labgene Scientific, Switzerland) and the dry weights were calculated. The dry cell mass was disrupted by resuspending pellets in 2 mL of 14% HNO_3_, then heated to 95°C for 20 min. Samples were centrifuged at 13,200 *g* for 30 min, the supernatants were collected and an internal standard indium (In) was added to the samples. Metal contents of these samples were analyzed in an iCAP ICP-MS (Thermo Scientific, U.S.A.). Results were showed as ng of Fe and Ru per mg dry weight of cells. All data were evaluated with at least three independent biological experiments.

### Real-time quantitative PCR (RT-qPCR)

Total RNA was extracted from *S. pneumoniae* D39 strain with and without R-825 (in sub-MIC) treatment by TRIZOL method according to the manufacturer's manual, and quantified by Nanodrop 2000 spectrophotometer. Any genomic DNA was removed using RNase-free DNase I. cDNA synthesis was performed using 1 µg of total RNA and iScript Reverse Transcriptase (Bio-Rad) according to the manufacture's instruction. RT-qPCRwas carried out using EvaGreen Dye (Bio-Rad) in a Miniopticon RT-qPCR System (Bio-Rad). The cycle threshold (Ct) value was measured; relative quantification of specific gene expression was calculated using the 2^−ΔΔCt^ method, with the 16S rRNA as the reference gene. Genes with a two-fold or greater difference in expression level relative to control were considered significant. The primer sequences used for RT-qPCR are shown in Table 1.

**Table 1:** The primer sequences used for RT-qPCR experiments.

Primer	Sequence (5′–3′)
16S rRNA-F	5′-CTGCGTTGTATTAGCTAGTTGGTG-3′
16S rRNA-R	5′-TCCGTCCATTGCCGAAGATTC-3′
PiaA-F	5′-TAGTCAGACAGAGACCAGT-3′
PiaA-R	5′-CTTTCATAGAACCAACATT-3′
PiuA-F	5′-ATTTGACGATTTGGATGGACTT-3′
PiuA-R	5′-GATTTGTATGCTGCTACAGGAG-3′
PitA-F	5′-ATGACTGTTGGTCTCTCTT-3′
PitA-R	5′-TTGTTTTAGCATTTTTACG-3′

### Cloning and purification of PiuA protein

The *piuA* gene without the N-terminal lipoprotein signal sequence was PCR amplified from *S. pneumoniae* D39 genomic DNA with the forward primer 5′-CCGCCGGAGCTCTCTTCTAATTCTGTTAAAAA-3′ and the reverse primer 5′-GCCGCCGAATTCTTATTTCGCATTTTTGC-3′, creating the SacI and EcoRI restriction sites (underlined). The PCR product was digested with SacI and EcoRI and ligated into the expression vector pBAD/HisA to generate pBAD-PiuA. The construct was transformed into *E. coli* TOP10 for high-level expression of recombinant protein. The transformants were incubated at 37°C with vigorous shaking in LB medium when the OD_600_ reached at 0.8, followed by induction with 0.05% L-arabinose for 6 h. Harvested cells were lysed by sonication for 30 min (5 s on/5 s off, on ice), the supernatant was collected by centrifugation at 4 °C, 10,000 *g* for 30 min and the protein was then isolated by using Ni-NTA His-bind Resin (1.5 mL, Qiagen). Fractions containing His6-PiuA protein were harvested and verified with SDS-PAGE and Western blotting. The His-tag was cleaved with entorokinase for 24 h at room temperature, and then removed by Ni-NTA to produce purified PiuA protein. The purity of the purified protein was examined by SDS-PAGE. The identity of the PiuA protein was further confirmed by using ABI-4800 plus MALDI TOF/TOF mass spectrometer according to a previously described method [16]. Proteins were identified by the MASCOT search engine (V2.1) against NCBI *S. pneumoniae* D39 protein database based on the MS and MS/MS spectra, protein identifications with Mascot scores C. I. % >95 were considered significant. PiaA protein (the forward primer piaA-F: 5′-GCGAGCTCGAGACCAGTAGCTCTGCTC-3′, and the reverse primer piaA-R: 5′-CGCCGCGAATTCTTATTTCAAAGCTTTTTG-3′) and PitA protein (the forward primer pitA-F: 5′-GCGAGCTCATGACTGTTGGTCTCTCTTA-3′, and the reverse primer pitA-R: 5′-CGCCGCGAATTCTTACTGTTTAGATTGGATAT-3′) were also cloned and purified using the similar procedure.

### Immunization experiments and Western blotting

Purified His6-PiaA, His6-PiuA and His6-PitA proteins were used as antigens for the immunization experiments as previously described in the literature to generate multicolon antibodies [17]. The specificities of the antibodies were detected with Western blotting using purified proteins and whole-cell lysates of wild type and *piaA*-, *piuA*- and *pitA*- mutant D39 strains.

For Western blotting analysis, untreated and R-825-treated (sub-MIC) *S. pneumoniae* D39 were harvested by centrifugation at 6,000 *g* for 10 min at 4°C when the absorbance reading of 0.6 at 600 nm was reached. Then pellets were washed three times with 1×PBS and disrupted by sonication to extract proteins, the concentrations of the cellular proteins were measured by Bradford assay. The protein extracts were separated by 12% SDS–PAGE and then electroblotted onto polyvinylidene fluoride membranes. The protein expressions of PiaA, PiuA and PitA were detected with anti-PiaA, -PiuA and -PitA antibodies and quantified using ImageMaster 2D Platinum 6.0. Total proteins separated by SDS-PAGE and stained with Coomassie brilliant blue R250 were used as the loading control.

### Fluorescence spectroscopy of apo-PiuA protein titrated with R-825

Fluorescence measurements were performed in a Hitachi F7000 spectrofluorophotometer. Fluorescence emission spectra were recorded from 290 to 450 nm after exciting at 280 nm. Both slit widths of excitation and emission beams were 5 nm. Spectra were acquired for 2 µM apo-PiuA (20 mM Tris-HCl, 100 mM NaCl, pH 7.4) with varying concentrations of R-825 (from 1.2 to 19.6 µM) and ferrichrome (from 0.4 to 4.0 µM). Vancomycin (from 1.2 to 19.2 µM) was also titrated to the apo-PiuA solution as a negative control. Relative changes in fluorescence emission (△F) at 343 nm during the titration verses the concentrations were fitted to a titration curve. The data were analyzed with Hill plot equation in Origin 8.5 to acquire the affinity constants (Ka).

## Results

### Ru(II) complex R-825 possesses antibacterial activity against *S. pneumoniae*

**Figure 2:**
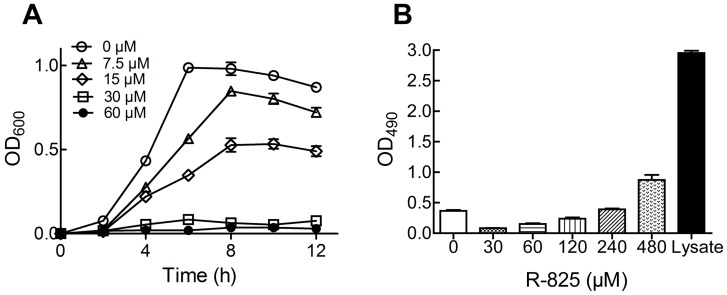
Effect of R-825 on *S. pneumoniae* and human A549 cell growth. (A) R-825 inhibited *S. pneumoniae* growth in a concentration-dependent manner. (B) R-825 showed little cytotoxicity to human cells. Determination of the cytotoxicity against A549 cell line was performed by incubating the cells with R-825 for 48 h using LDH kit, cell lysate was used as positive control. The data shown represent the mean of three experiments; error bars indicate SEM.

To investigate the antibacterial activity of R-825, we tested its effects on the growth of *S. pneumoniae* D39 strain in batch cultures, and determined its MIC value for *S. pneumoniae*. As shown in Figure 2A, R-825 can inhibit the bacterial growth in a concentration-dependent manner. At the concentration of 30 µM (MIC), R-825 completely suppressed the growth of *S. pneumoniae*. The sub-MIC, 15 µM, was selected to treat *S. pneumoniae* for the following real-time quantitative PCR and ICP-MS assays. MIC determination for the mutant strains was also performed and the results are listed in Table 2. Notably, *piuA*- mutant has a MIC  =  60 µM, double of those for the wild-type and other mutant strains.

**Table 2:** Minimal Inhibitory Concentrations (MICs) and Diameter of Growth Inhibition Zones (mm) of R-825 against WT *S. pneumoniae* D39, *piaA-*, *piuA*- and *pitA*- mutant strains.

Bacteria	MICs (µM)	Diameter of Growth Inhibition Zones (mm)
WT *S. pneumoniae* D39	30	16
*piaA*- mutant	30	16
*piuA*- mutant	60	10
*pitA*- mutant	30	14

### R-825 is not toxic to human A549 cells

The cytotoxic activity of R-825 against human A549 cells was evaluated by using LDH assay kit, with whole cell lysate as a positive control. As shown in Figure 2B, the viability of A549 cells had not significant changes under the 48 h treatment of R-825 with up to 480 µM, a concentration substantially higher than the corresponding MIC value for the bacterium. Results demonstrated that R-825 has very low toxicity to human cells, implicating its high selective toxicity toward bacteria over human host.

### R-825 reduces bacterial iron content and up-regulates PiaA and PiuA

**Figure 3:**
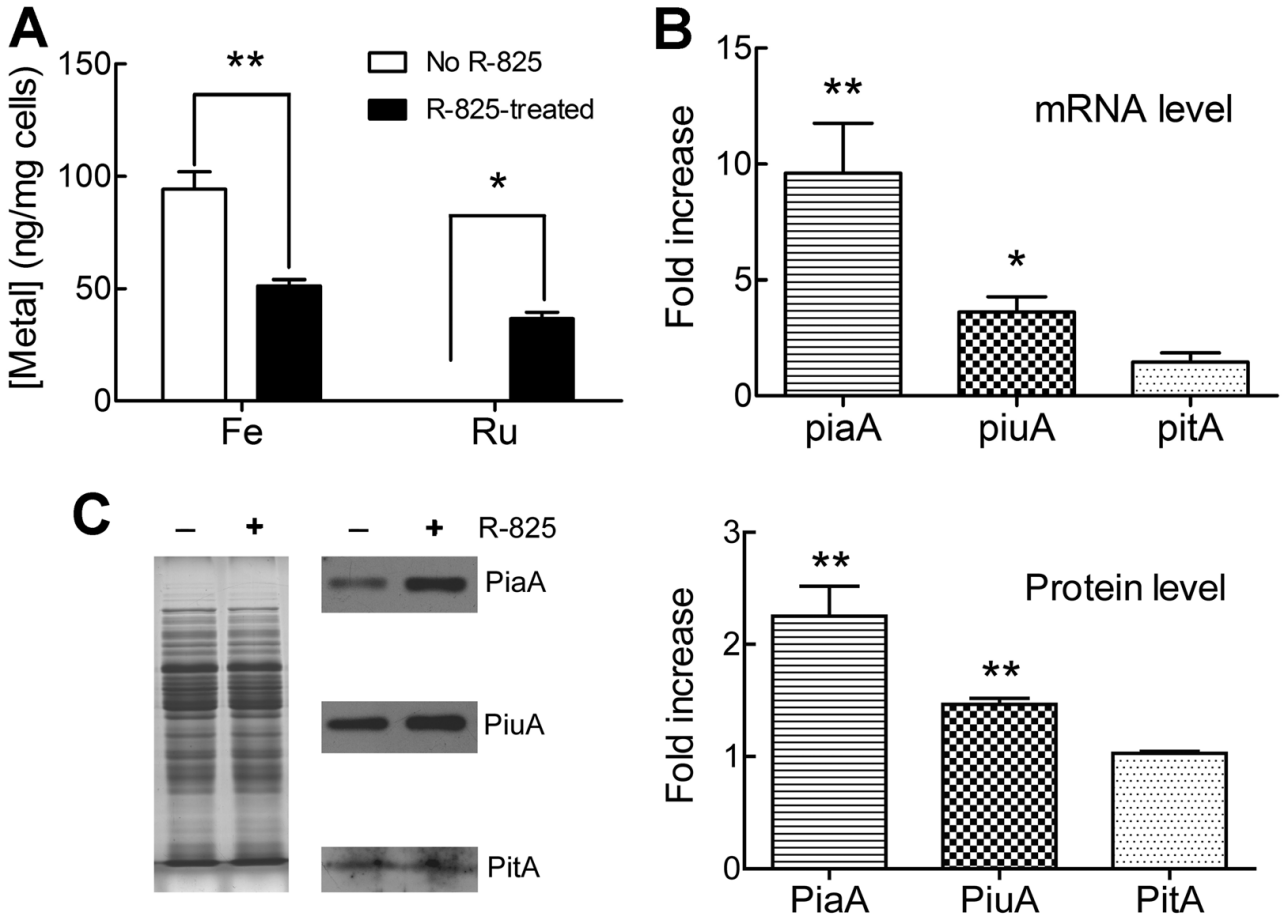
R-825 reduced iron uptake and stimulated the expressions of PiaA and PiuA in *S. pneumoniae*. (A) The cellular iron and ruthenium contents of the wild-type *S. pneumoniae* with and without R-825 treatment. (B) The increase of mRNA expression levels of *piaA*, *piuA* and *pitA* in the presence of 15 µM R-825, as compared with their expressions in the absence of R-825. Data were normalized with housekeeping gene 16S rRNA (mean ± SEM, n  =  3, **p < 0.01, *p < 0.05 versus the untreated control). (C) The comparison of the protein expression levels in the bacterium with and without R-825 treatment. Whole cell proteins were used as loading control for the Western blotting.

To determine whether R-825 can compete with iron for the uptake system by *S. pneumoniae*, we measured the intracellular iron and ruthenium levels in the bacterium with and without R-825 treatment by using ICP-MS analysis. The results are shown in Figure 3A. As compared with control, the treatment with R-825 in its sub-MIC significantly decreased the iron concentration and correspondingly increased the ruthenium concentration in wild-type *S. pneumoniae*.

Previous studies have shown that many metal ion transporters are regulated by their substrates [18]–[20]. To investigate whether R-825 regulates iron-uptake proteins, we monitored the mRNA expression levels of lipoproteins PiaA, PiuA and PitA, the three surface iron chelates in the known iron-uptake ABC transporter systems in *S. pneumoniae* [6], [7]. We observed that R-825 treatment up-regulated *piuA* and *piaA* gene expressions, but exhibited no significant impact on *pitA* expression, as shown in Figure 3B. Western blotting was also performed to measure the protein expression levels after R-825 treatment. As shown in Figure 3C, R-825 stimulated the expression of PiuA and PiaA but exerted no effect on PitA expression.

To confirm the interference of R-825 with the iron uptake system, we also investigated the protein expression level in the bacteria cultured with iron replete and restricted medium. As shown in Figure S1 in File S1, upon iron starvation, the expression level of PiaA is significantly increased, and PiuA is slightly increased while PitA is unchanged in wild-type D39 strain. However, the expression levels of both PiuA and PitA are evidently up-regulated in *piaA*- mutant strain upon iron starvation. These results are basically consistent with the protein change tendency upon Ru-825 treatment. These consistent observations suggest that R-825 may enter *S. pneumoniae via* PiaABC or PiuABC iron-uptake systems.

### R-825 is uptaken *via* ferrichrome-transport system

To determine whether R-825 would be taken up by *S. pneumoniae* via PiaABC (for heme uptake) or PiuABC (for ferrichrome uptake) system, we individually deleted *piaA* and *piuA* genes in the bacterium to construct the *piaA*- and *piuA*- single mutant strains, in which heme- and ferrichrome-binding ability was respectively impaired in the bacterium, and verified the effects using Western blotting (figure S2 in File S1). The sensitivity of the mutant strains to R-825 was compared to that of wild-type *S. pneumoniae* by measuring the growth inhibition zones and MIC values corresponding to R-825 treatment. As comparison, *pitA*- mutant was also constructed and tested (figure S2 in File S1).

**Figure 4:**
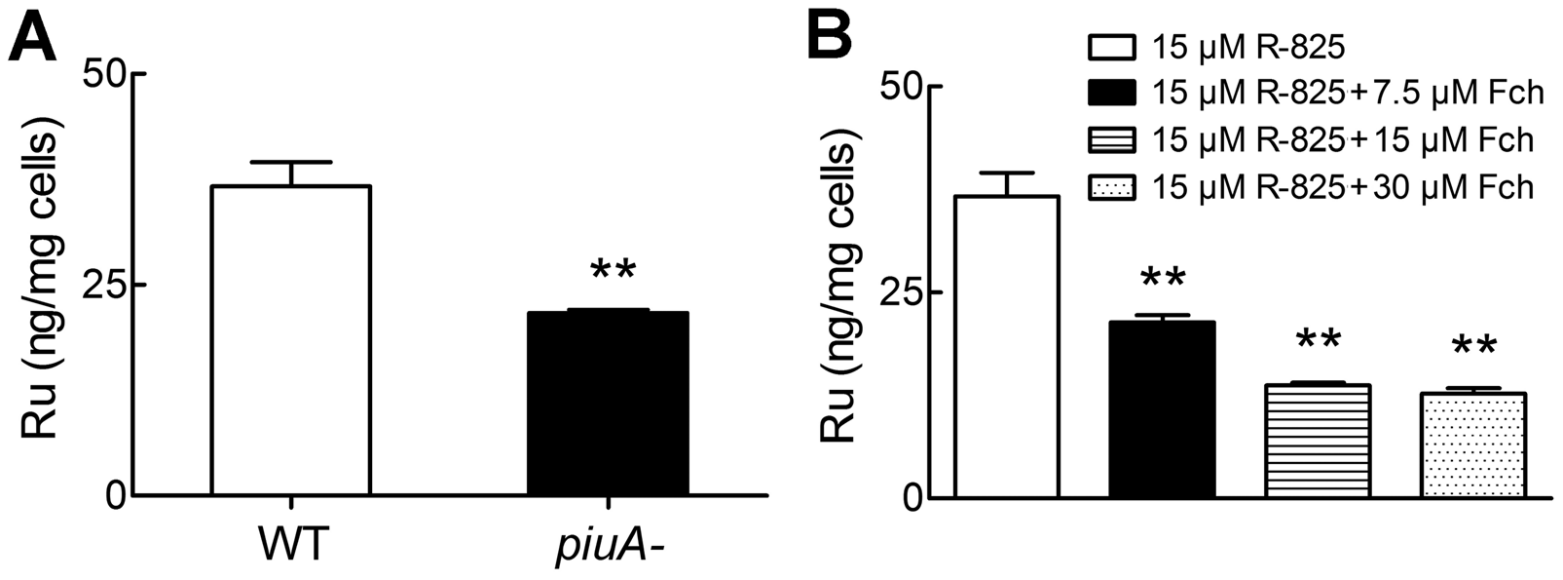
Cellular ruthenium concentrations in R-825 treated *S. pneumoniae* and with ferrichrome (Fch) competition as determined by ICP-MS. (A) Ruthenium contents in wild-type *S. pneumoniae* and in *piuA*- mutant as treated with 15 µM R-825. (B) Ruthenium contents in wild-type *S. pneumoniae* in the addition of increasing ferrichrome. Results are representative of the mean±SEM from three independent experiments (*p<0.01).

Our experimental results are shown in Table 2. As compared with wild-type *S. pneumoniae*, both *piaA*- and *pitA*- mutants exhibited no significant difference in growth inhibition zones and MICs. In contrast, *piuA*- mutant strain displayed much smaller growth inhibition zone with a doubled MIC value, indicating that the *piuA*- strain is resistant to R-825 (Table 2). This suggests that the antibacterial activity of R-825 is largely related to PiuA but not PiaA and PitA; without PiuA, the uptake of R-825 may be impaired in the *piuA*- mutant strain. To confirm this hypothesis, we detected the intracellular ruthenium levels in the *piuA*- mutant and wild-type strains under R-825 treatment. As observed in Figure 4A, the level of intracellular ruthenium in *piuA*- mutant strain was substantially reduced to half content of the wild-type bacterium.

### Ferrichrome but not iron ion and hemin rescues the bacterial growth suppressed by R-825

**Figure 5:**
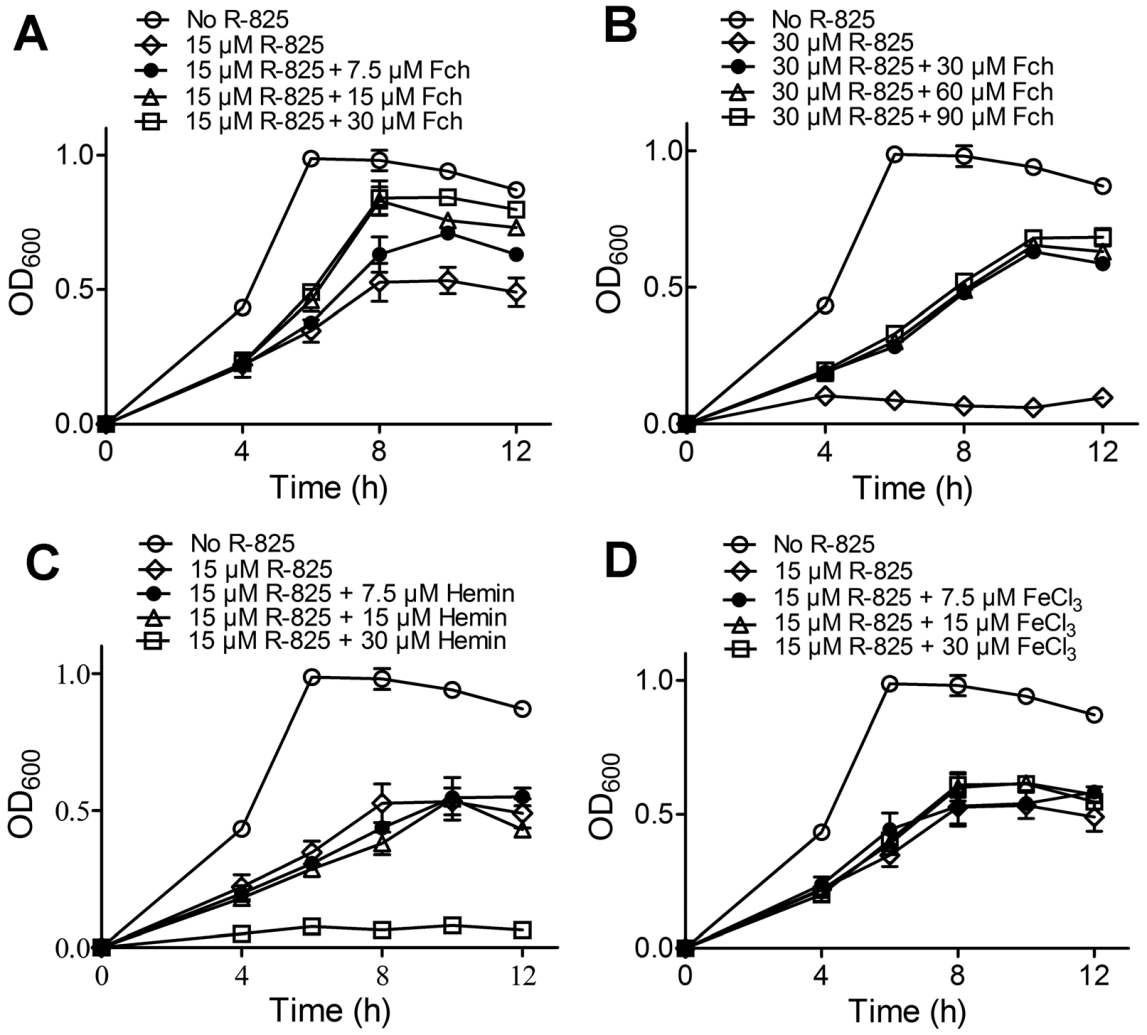
Ferrichrome, but not hemin or FeCl_3_ rescued the bacterial growth suppressed by R-825. (A) Sub-MIC treated *S. pneumoniae* with increasing amounts of Fch. (B) MIC-treated *S. pneumoniae* with increasing amounts of Fch. (C) Sub-MIC treated *S. pneumoniae* with increasing amounts of hemin. Hemin itself at ≥30 µM has toxicity to the bacterium, causing the further inhibition of the bacterial growth. (D) Sub-MIC treated *S. pneumoniae* with increasing amounts of FeCl_3_.

The above experiments indicated that R-825 may be taken up by *S. pneumoniae* through PiuABC, the ferrichrome transport system. Accordingly, ferrichrome should be able to compete with R-825 for the uptake and antagonize the antimicrobial activity of R-825. We therefore examined the antimicrobial activity of R-825 against *S. pneumoniae* in the presence of increasing concentrations of ferrichrome, hemin and FeCl_3_. As shown in Figure 5, ferrichrome addition indeed reversed the growth-inhibitory effects of R-825 in a dose-dependent manner with either sub-MIC or MIC treatments (Fig. 5A and 5B, respectively). When the molar ratio of ferrichrome to R-825 reached higher than 2:1, the maximum OD_600_ of bacterial growth could be restored to near normal level. In contrast, adding either hemin (Fig. 5C) or FeCl_3_ (Fig. 5D) could not rescue the bacterial growth inhibited by R-825. The complete inhibition observed in Figure 5C with 30 µM hemin addition was due to the toxicity of hemin itself to the bacterium.

We also measured the bacterial ruthenium contents in response to the ferrichrome addition in Figure 5A. As shown in Figure 4B, ferrichrome addition gradually reduced the cellular concentration of ruthenium while rescuing the bacterial growth (Fig. 5A). This observation indicated an actual competition between ferrichrome and R-825 for the bacterial uptake. These results all together confirmed that R-825 enters *S. pneumoniae* indeed *via* ferrichrome-uptake pathway PiuABC, rather than PiaABC and PitABC transport systems.

### R-825 can bind to PiuA protein *in vitro*

**Figure 6:**
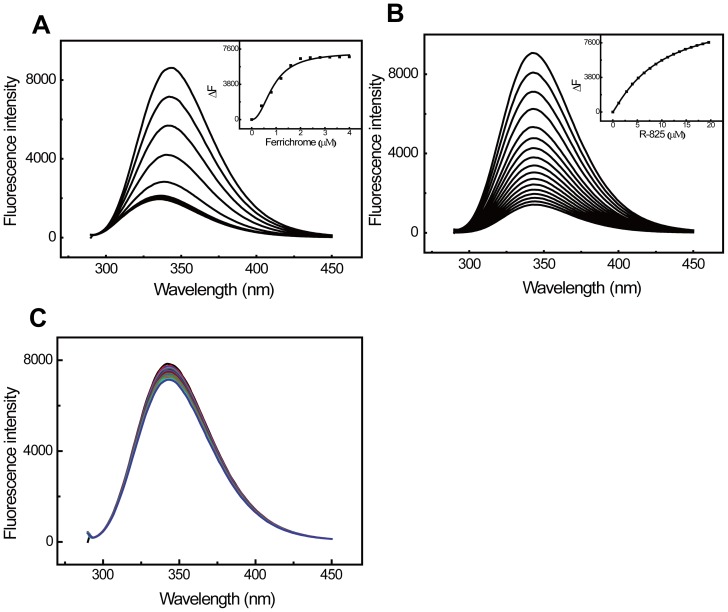
R-825 binds to PiuA protein *in vitro*. Fluorescence spectra of 2 µM apo-PiuA protein in 20 mM Tris-HCl (pH 7.4) titrated with aliquot ferrichrome (A), R-825 (B), or vancomycin (C). The inserted binding isotherm curves were built from the relative change values of fluorescence intensity (ΔF) at 343 nm versus concentrations. The inner panels are the curve-fitting analyses using Hill plot in the program Origin 8.5.

The binding between R-825 and PiuA protein was tested to validate the interaction of R-825 with PiuABC systems. For comparison, the binding between ferrichrome/vancomycin and PiuA was also determined. The quenching fluorescence spectra of apo-PiuA upon titration with the chemicals are shown in Figure 6; both R-825 and ferrichrome have a similar binding pattern with PiuA protein, while vancomycin as a negative control almost does not bind to PiuA protein. When the step-wise fluorescence quenching versus chemical concentration data were curve-fitted to Hill plot equation 1, the binding constants were determined to be (0.30 ± 0.18)×10^6^ M^−1^ and (1.12 ± 0.28)×10^6^ M^−1^ for R-825 and ferrichrome, respectively. These results revealed that both R-825 and ferrichrome could specifically bind to PiuA, with ferrichrome having a higher binding affinity than R-825 for PiuA.

## Discussion

Bacterial resistance to antibiotics is becoming a significant threat to global public health [21]. There is an urgent need to develop novel antimicrobial drugs with an action mode different from the current antibiotics. The bacterial cell membrane demonstrates decreased permeability, serving as a barrier to limit the intracellular access of substances such as antibiotics by passive diffusion. Reduced membrane permeability is one of the main mechanisms for antibiotic resistance (reviewed in [22]). A strategy to circumvent the 'impermeability' resistance problem is to target the iron-transport systems for the drug delivery into the bacterial cells. Since the iron-transport systems are widespread among bacteria and work as active channels to transfer iron, some studies had explored antibacterial compounds including Ga(NO_3_)_3_ [23], DFO-Ga [24], non-iron metalloporphyrins (MPs) [25], and albomycin [8], enterobactin-cargo conjugates [26] as ‘Trojan horses’ to enter the bacteria through the iron-uptake systems.

In our long-term research on the mechanism of iron acquisition in bacteria, we understand that most iron obtained by bacteria is in the form of siderophores or heme since free iron ion is severely restricted in the host. Accordingly, blocking or interfering with either the heme- or siderophore-uptake pathway would be an effective approach to limit the acquisition of iron, one of the crucial elements for bacterial growth and survival. Here we selected our previously synthesized ruthenium(II) complex R-825 (Fig. 1) for the experiment, with an expectation that this compound can compete with either heme or ferrichrome to bind to the iron-receptors PiaA or PiuA, reduce the availability of iron to the bacteria and thus suppress the bacterial growth. Both ruthenium and iron are the members of VIII family in the periodic table, sharing chemical similarities in terms of coordination and binding with ligands. More importantly, Ru(II) is not toxic to the human body, making it an excellent candidate for antimicrobial drug development. Our current experiments demonstrated that R-825 indeed has selective antimicrobial activity against *S. pneumoniae*, with no evident toxic effects towards human A549 cells even in a concentration significantly greater than the corresponding MIC value (Fig. 2).

As expected, we detected that R-825 can be internalized by *S. pneumoniae*, accompanying with the decrease of bacterial iron uptake (Fig. 3A). Correspondingly, the expression of lipoproteins PiaA and PiuA in the bacterium was stimulated to compensate for the decreased iron availability under R-825 competition (Fig. 3B&C). Obviously, PiaA and/or PiuA iron-uptake systems were involved and thus we constructed *piaA*- and *piuA*- gene deletion mutant strains for further tests. Based on the MIC and growth inhibition zones assays (Table 2), we observed that only *piuA*- mutant showed an increased resistance to R-825, accompanying with a significant decrease in the content of cellular ruthenium in the mutant (Fig. 4A). This means that, without PiuA, less R-825 can be uptaken into cells and thus the bacterium became resistant to the drug. In other words, R-825 may be internalized by the bacterium mainly *via* PiuABC pathway.

Among the three iron-transport systems in *S. pneumoniae*, PiuABC system mainly conveys ferrichrome and its analogues into cells with the interaction between PiuA and ferrichrome [8]. Our experiments validated that R-825 can also interact with PiuA *in vitro*, in a binding pattern similar to that of PiuA-ferrichrome interaction (Fig. 6), suggesting that an active competition between R-825 and ferrichrome may occur for PiuA binding *in vivo*. Correspondingly, adding ferrichrome to the medium should compete with R-825 for the binding to PiuA, decrease the ruthenium uptake (Fig. 4B) and thus antagonize the antibacterial activity of R-825. Our competition experiments support this observation, in which adding ferrichrome can rescue the R-825-suppressed bacterial growth in a dose-dependent manner while adding hemin or FeCl_3_ had no affect (Fig. 5). We can therefore conclude that R-825 interferes with the ferrichrome-uptake system by competing with ferrichrome for PiuA binding, reduces the iron internalization and thus suppresses the bacterial growth.

The fact that ruthenium compounds have antimicrobial activities has been recognized recently [27]–[31]. In particular, Keene and his co-workers characterized the susceptibility and cellular uptake of inert Ru(II) complexes [30], [31] and demonstrated that di-nuclear Ru(II) complexes may enter eukaryotic cells by passive diffusion, while mononuclear complexes may have a different mode of action. This observation echoes our current finding that mononuclear Ru(II) complex R-825 is mostly uptaken into cells *via* the active ferrichrome-transport pathway. Certainly, this active transportation may not be the only way for R-825 to enter the bacterium, as attested by the fact that certain amounts of ruthenium were still detected in the *piuA*– mutant strain (Fig. 4A). On the other hand, our R-825 has a lower affinity than ferrichrome for PiuA binding (Fig. 6), suggesting that this compound can be further modified in terms of its structure and lipophilicity to enhance the ability to compete with ferrichrome and thus to optimize its antibacterial activity against *S. pneumoniae*.

**Figure 7:**
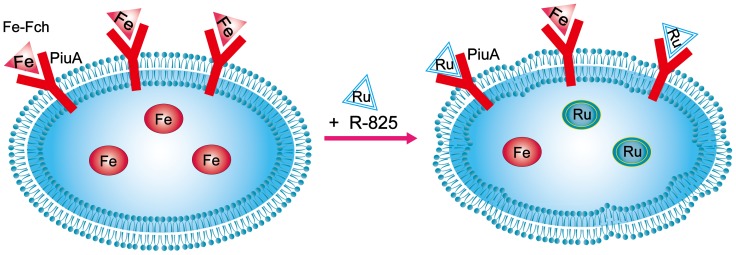
A model to summarize the proposed mechanism of Ru-complex action on the bacteria. R-825 is uptaken by *S. pneumoniae via* ferrichrome-transport pathway, reducing the competitive iron uptake into cells and thus suppressing the bacterial growth.

In summary, we used a Ru(II) complex R-825 to chemically compete with iron compounds for binding to the iron-binding ligands in the iron-transport systems in *S. pneumoniae*. By performing various experiments, we demonstrated that R-825 can be transported into the bacterium *via* ferrichrome-transport pathway, competitively reducing iron uptake into cells and thus suppressing the bacterial growth (Fig. 7). Since the ferrichrome-uptake pathways are widely spread among bacteria, R-825 and derivatives may represent a new candidate class of antimicrobial agents for further development.

## Supporting Information

File S1 **Figure S1.** The relative levels of PiaA, PiuA and PitA proteins in the iron replete or restricted conditions. **Figure S2.** The constructed *piaA*-, *piuA*- and *pitA*- mutant strains were verified by Western blotting and the purity of PiuA protein was verified by SDS-PAGE.
(DOCX)
