# Supplementary material for: Chemical Interference with Iron Transport Systems to Suppress Bacterial Growth of Streptococcus pneumoniae
Source: PLoS One. 2014 Aug 29;9(8):e105953. doi: 10.1371/journal.pone.0105953 (PMC4149436; doi:10.1371/journal.pone.0105953)
Supplement: File S1 — Figure S1. The relative levels of PiaA, PiuA and PitA proteins in the iron replete or restricted conditions. Figure S2. The constructed piaA-, piuA- and pitA- mutant strains were verified by Western blotting and the purity of PiuA protein was verified by SDS-PAGE. (DOCX) [file pone.0105953.s001.docx]

**Supporting Information**


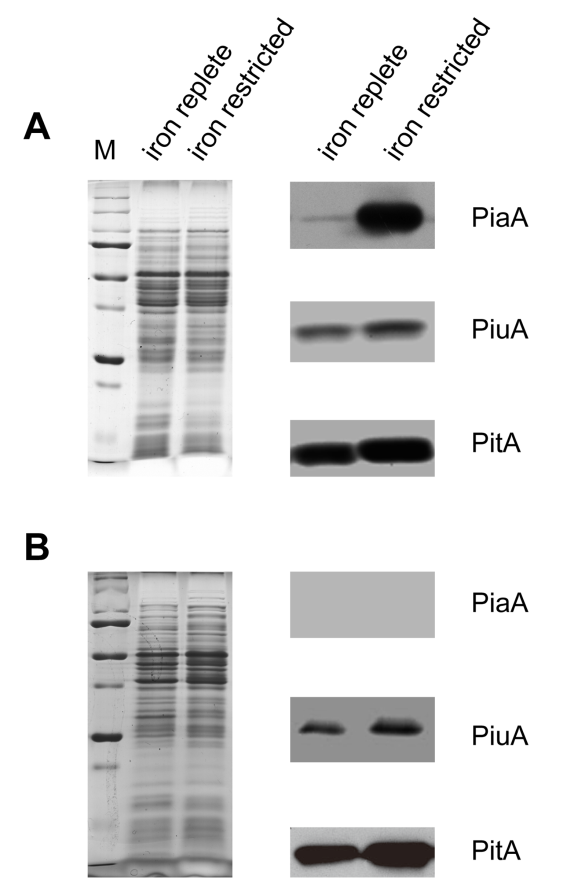


**Supplementary Figure S1.** The relative levels of PiaA, PiuA and PitA proteins in the iron replete or restricted conditions. Western blotting showed the relative levels of PiaA, PiuA and PitA in the wild-type (A) and *piaA-*mutant (B) D39 strain grown under the iron replete or restricted conditions, total proteins as a control.


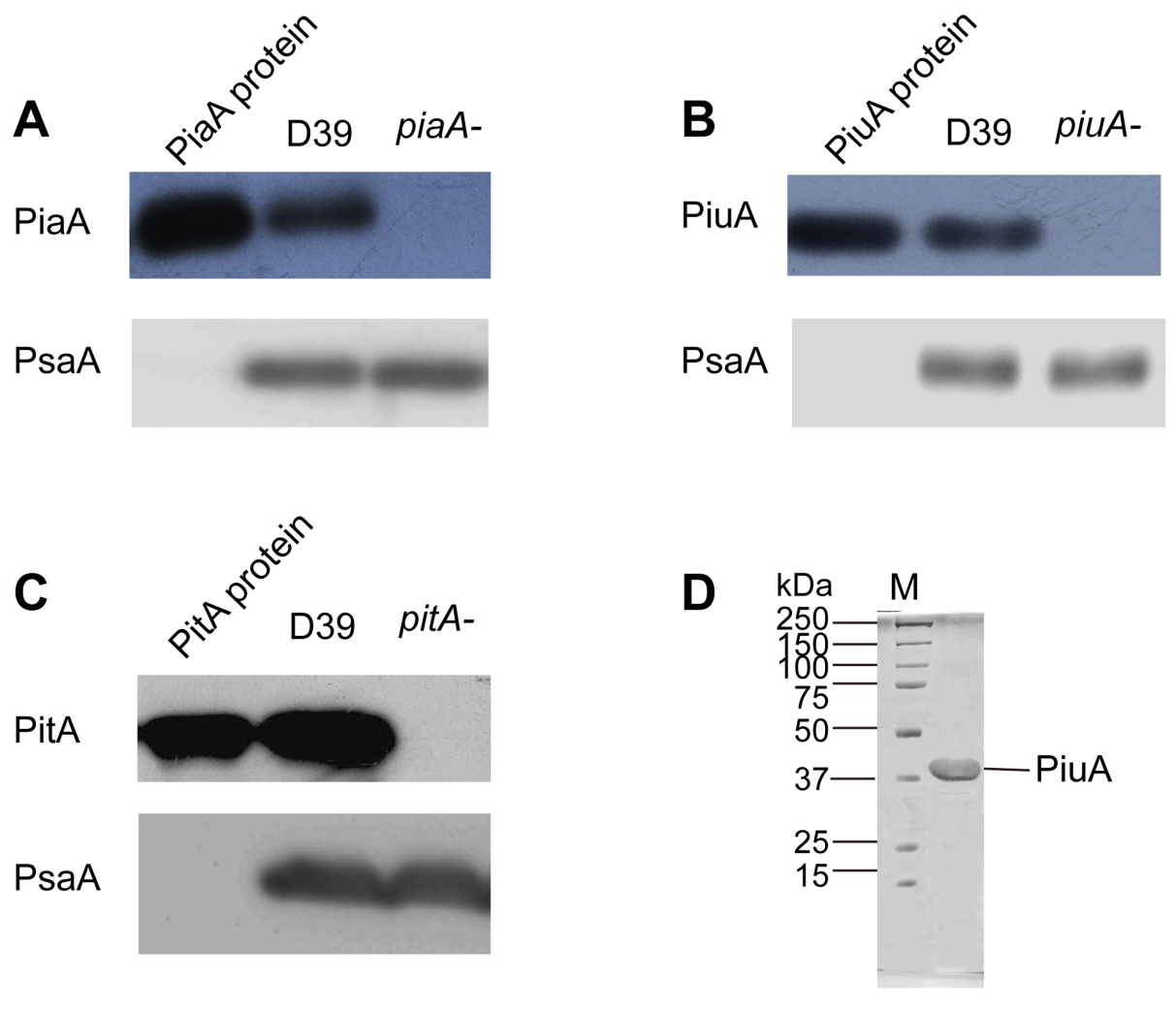


**Supplementary Figure S2**. The constructed *piaA*-, *piuA*- and *pitA*- mutant strains were verified by Western blotting (A-C) and the purity of PiuA protein was verified by SDS-PAGE (D). PsaA protein was using as loarding control in Western blotting.
